# Commentary: First master the fundamentals

**DOI:** 10.1016/j.xjtc.2021.07.003

**Published:** 2021-07-10

**Authors:** Christopher E. Mascio, Vinay Badhwar

**Affiliations:** Department of Cardiovascular and Thoracic Surgery, West Virginia University, Morgantown, WVa

**Figure:**
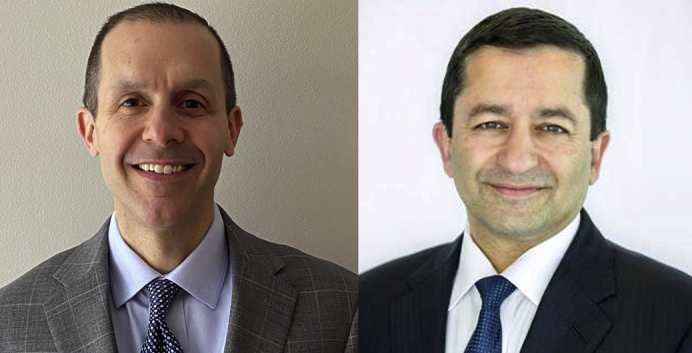
Christopher E. Mascio, MD (*left*), and Vinay Badhwar, MD (*right*)

**Central Message**

BAV morphology and the best surgical therapy for each phenotype is complex and evolving. This video provides an important educational review of the various BAV types.

See Article page 44.

Woo and colleagues^1^ present a video demonstrating relationships of circumferential angles, cusp fusion length, and commissural fusion height in normal aortic valves, type 1 bicuspid aortic valves (BAV), and type 0 BAV, as classified by Sievers and Schmidtke.^2^ Although actual presentation of pathology is not an evolving continuum in a single patient, this video presents this information in sequential fashion commencing with the 120° commissural angle, 20 mm commissural height, and 0 mm fusion length of a normal tricuspid aortic valve, and concludes with the 180° commissural angle, 0 mm commissural height, and 20 mm fusion length of a type 0 BAV. Much has been discovered about the best therapies for BAV over the past decade, and the treatment algorithm today is quite sophisticated. As with any complex task, mastering the fundamentals is critical to success. This video provides a visual representation of the various anatomic phenotypes encountered by the cardiovascular surgeon, and in turn, engenders an essential understanding critical for proper treatment.

Pathologies related to BAV are among the most common congenital cardiac anomalies, with an incidence of up to 2% in the general population.^3^^,^^4^ It has also been associated with dilatation of the ascending aorta and subsequent aneurysm and/or dissection formation.^5^ This information, along with advanced surgical techniques and a better understanding of the structure and function of the aortic valve, prompted Sievers and Schmidtke^2^ to describe a classification system for BAV. He described 3 types: type 0 (0 raphe), type 1 (1 raphe), and type 2 (2 raphes). As has been illustrated, these different BAV entities were not all best treated with the same surgical therapy; for example, the Ross procedure and valve replacement in type 0 BAV is more challenging, requiring a circular proximal suture line and more attention to placement of the coronary buttons.^2^

This growing knowledge of BAV has led to more elaborate discussion about interventions on this disease entity. Jahanyar and colleagues^6^ describe the varying surgical approaches recommended for different phenotypes. Their repair-oriented classification includes symmetric, asymmetric, and very asymmetric types. The repairs are quite different for each type and range from cusp plication and 180° repair (symmetric) to commissurotomy with commissure resuspension and patch reconstruction (very asymmetric). Similarly, de Kerchove and colleagues^7^ describe symmetric (type A) to asymmetric (type C) phenotypes, and propose different repairs based on certain anatomic characteristics, including commissure orientation, length of fusion, and nonfunctional commissure height. Now, many patients with insufficiency due to BAV can undergo repair with adjunctive remodeling ring annuloplasty, and via a less-invasive manner.^8^

We commend the authors on this succinct and elegantly presented introduction to BAV pathology. This video could be the first step of creation of an important resource for trainees and early career cardiovascular surgeons.
